# Successful Mitral and Tricuspid Valve Repair for Dextrocardia With Situs Inversus Totalis

**DOI:** 10.1016/j.atssr.2022.10.018

**Published:** 2022-11-03

**Authors:** Ryo Kawasumi, Satoshi Kainuma, Koichi Toda, Daisuke Yoshioka, Masashi Kawamura, Tetsuya Saito, Takuji Kawamura, Ryohei Matsuura, Shigeru Miyagawa

**Affiliations:** 1 Department of Cardiovascular Surgery, Osaka University Graduate School of Medicine, Suita, Osaka, Japan

## Abstract

Dextrocardia with situs inversus is a rare congenital anomaly characterized by a right-sided heart apex and inversely rotated abdominal viscera. It is often autosomal recessive and involves 1 child in every 10,000 births. We report a case of dextrocardia with situs inversus totalis in a patient who underwent mitral valve repair through an extended transseptal approach for clinically relevant mitral regurgitation secondary to Barlow disease. The operation was successfully performed without complications, and the postoperative course was uneventful.

Dextrocardia is a rare cardiac condition in which the heart is located toward the right side of the chest. Dextrocardia with situs inversus totalis, which involves mirror-image transposition of both thoracic and abdominal viscera across the left-right axis of the body, is a rare anomalous entity affecting 1 or 2 individuals per 10,000 population.^1^ Some cases can be combined with primary ciliary dyskinesia or other genetic mutations, but most of the cause remains unknown.^2^ We report successful mitral and tricuspid valve repair and atrial ablation procedure in a patient with dextrocardia with situs inversus totalis.

A 75-year-old woman diagnosed with dextrocardia and situs inversus totalis in childhood presented with clinically relevant mitral regurgitation (MR) and atrial fibrillation 2 years ago. She had been in good health for 2 months before admission for dyspnea on exertion during light physical activity. On admission, electrocardiography revealed atrial fibrillation with a heart rate of 76 beats/min; chest radiography showed that the heart was directed to the right with cardiomegaly. Laboratory findings were within normal limits, except for elevated serum brain natriuretic peptide level (230.8 pg/mL). Transthoracic echocardiography revealed normal left ventricular dimensions and systolic function with an ejection fraction of 76%. Transesophageal echocardiography revealed severe MR due to annular dilation and prolapse of the A2 segment with a myxomatous change in the entire valve leaflet, consistent with Barlow disease (Figure 1). The tricuspid valve was also substantially redundant, resulting in moderate tricuspid regurgitation. Computed tomography revealed dextrocardia with situs inversus, where the heart apex was right sided, and the viscera of the abdomen were inversely rotated (Figure 2). The anatomic position of the bilateral pulmonary veins was higher than usual; however, there was no interruption of the inferior vena cava.

**Figure 1:**
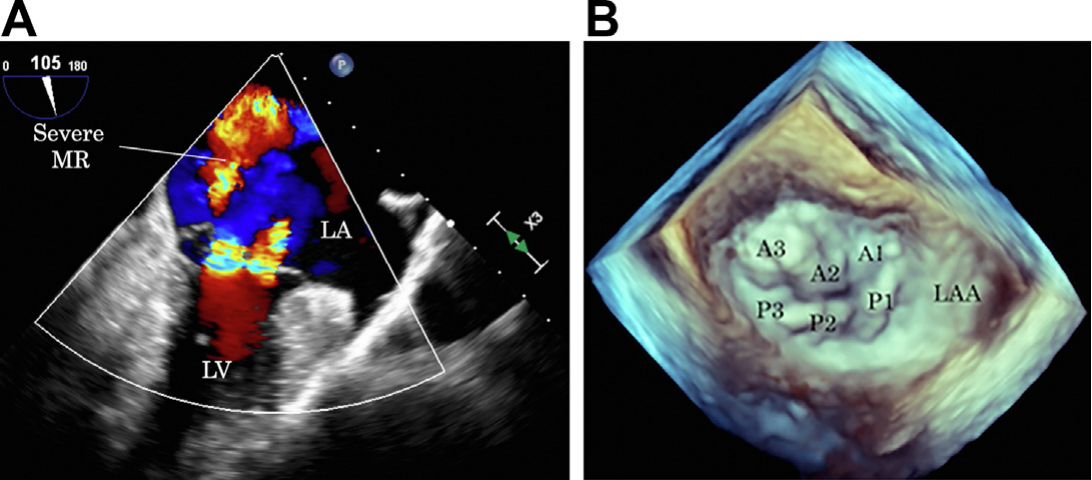
(A) Preoperative transesophageal echocardiography with color Doppler (midesophageal mitral commissural view) shows severe mitral regurgitation (MR) due to anterior leaflet prolapse. (B) Three-dimensional view of the mitral valve in dextrocardia as viewed intraoperatively by the surgeon standing on the left side of the patient. (LA, left atrium; LAA, left atrial appendage; LV, left ventricle; A1, the anterior segment of the anterior leaflet; A2, the middle segment of the anterior leaflet; A3, posterior segment of the anterior leaflet; P1, the anterior scallop of the posterior leaflet; P2, the middle scallop of the posterior leaflet; P3, the posterior scallop of the posterior leaflet.)

**Figure 2:**
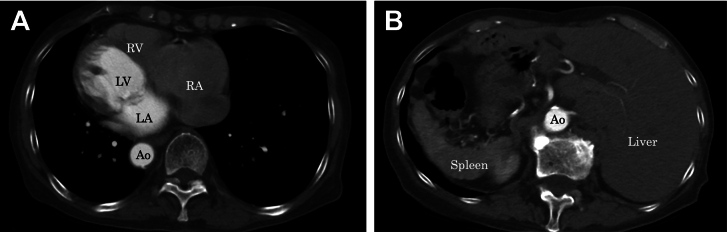
(A) Computed tomography imaging of the chest shows dextrocardia. (B) Computed tomography imaging of the abdomen shows situs inversus totalis. (Ao, aorta; LA, left atrium; LV, left ventricle; RA, right atrium; RV, right ventricle.)

Mitral and tricuspid valve repair with a maze procedure through median sternotomy was planned. A median sternotomy was performed, and dextrocardia with situs inversus was observed (Figure 3A). Next, cardiopulmonary bypass was established using ascending aortic cannulation and bicaval venous drainage. Left atrial appendage excision was performed using vascular staplers (Endo GIA; Medtronic). After aortic cross-clamping, the surgeon moved to the left. The right atrium was incised, and a right-sided maze procedure was performed with cryoICE (AtriCure). The interatrial septum was incised toward the left atrial roof, and the mitral valve was exposed (Figure 3B). The left side of the left atrium was then incised simultaneously, and a left-sided maze procedure using cryoICE was performed. Inspection of the mitral valve revealed A2, A3, and Pc prolapse with annular dilation. Therefore, adjustable artificial chordae were placed from the posteromedial papillary muscles to the leaflet of A3 and Pc, and a 30-mm MEMO 3D semirigid annuloplasty ring (LivaNova) was implanted. Saline testing revealed trivial MR. The tricuspid valve was exposed after closure of the interatrial septum, and prominent annular dilation was observed. Considering the mirror-image anatomy of the tricuspid valve and the position of Koch triangle in dextrocardia, tricuspid annuloplasty was performed with an inverted Carpentier-Edwards Physio tricuspid annuloplasty ring (Edwards Lifesciences). The patient was smoothly weaned off cardiopulmonary bypass. The postoperative course was uneventful, and echocardiography after the operation demonstrated mild MR. The patient was discharged on postoperative day 15 without complications. Echocardiography performed 1 year after the operation revealed no recurrence of the MR.

**Figure 3:**
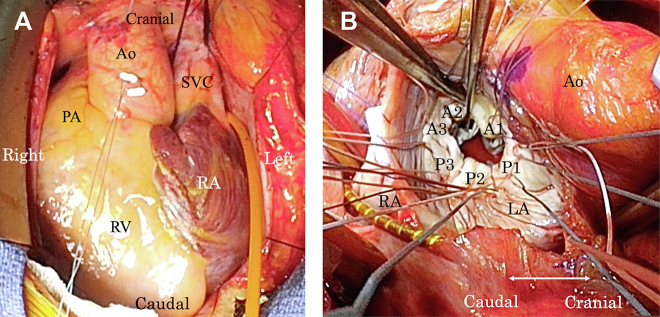
(A) Intraoperative view of the patient shows dextrocardia. (B) Intraoperative view of the mitral valve through the transseptal approach. (Ao, aorta; LA, left atrium; PA, pulmonary artery; RA, right atrium; RV, left ventricle; SVC, superior vena cava.)

## Comment

Dextrocardia with situs inversus totalis is a congenital abnormality that occurs at an incidence of 1 per 10,000 births. Dextrocardia with situs inversus totalis is associated with a high incidence of venous return malformations, such as an interruption of the inferior vena cava. Therefore, preoperative evaluation with computed tomography is important to obtain appropriate inferior vena cava cannulation and venous drainage of the liver.^3^ Valve operation for patients with dextrocardia is challenging because of its anatomic complexity. Previous cases have reported mitral valve operation using a variety of techniques ranging from conventional median sternotomy to minimally invasive techniques.^4^ The surgeon usually stands on the left side of the patient to have comfortable access to the mitral valve. A transseptal approach through a right atriotomy provides good exposure of the mitral valve in patients with dextrocardia.^5^ It provides wide vision of the mitral valve by extending the incision toward the left atrial roof and allowing access to the tricuspid valve. Other cases have reported mitral valve replacement for ischemic and rheumatic MR,^6^^,^^7^ whereas mitral valve repair using artificial chordae is feasible for degenerative MR in dextrocardia patients. In our case, we performed mitral valve repair using adjustable artificial chordae and mitral annuloplasty with a semirigid annuloplasty ring, as the usual procedure. The key to successful valve repair is good exposure and a detailed evaluation of the mitral valve. Tricuspid regurgitation, indicating the need for the operation in patients with dextrocardia, is rare. A previous case reported sutured tricuspid annuloplasty,^8^ but for tricuspid regurgitation due to a severely dilated tricuspid annulus, ring tricuspid annuloplasty was effective. The point to be noted in this technique is that we need to plant a partial tricuspid annuloplasty ring upside-down to avoid the conduction system in Koch triangle because of the mirror-image anatomy of the tricuspid valve in dextrocardia.

In conclusion, we describe a patient with dextrocardia with situs inversus who underwent successful mitral and tricuspid repair. A transseptal approach provided excellent exposure of the mitral valve, and tricuspid valve annuloplasty using an inverted partial tricuspid annuloplasty ring was useful in dextrocardia. Meticulous preoperative planning and accurate understanding of the anatomy are key to a successful operation and a good postoperative course.

## References

[1] Blegen H.M. (1949). Surgery in situs inversus. Ann Surg.

[2] Merel C.P., Amaia C.C., Simon E.F., Guy V., Clyde F. (2020). The genetics of situs inversus without primary ciliary dyskinesia. Sci Rep.

[3] Yokoyama Y., Satoh H., Abe M., Nagashima M., Kurata A., Higashino H. (2011). Cardiac surgery for annuloaortic ectasia and mitral regurgitation in an adult patient with dextrocardia. Gen Thorac Cardiovasc Surg.

[4] Pham C.V., Nguyen D.H., Vo A.T., Nguyen T.T., Phan L.H., Nguyen B.H. (2020). Minimally invasive mitral valve replacement and concomitant Cox-Maze IV procedure using radiofrequency energy in situs inversus totalis: a case report. Int J Surg Case Rep.

[5] Stiru O., Geana R.C., Ilie R.R. (2020). Transseptal approach for mitral valve replacement in dextrocardia with situs inversus totalis: a case report and review of the literature. Heart Surg Forum.

[6] Aggarwal P., Reddy R., Yashvinder Singh RS. (2019). Multivalvular rheumatic heart disease in a case of dextrocardia with situs inversus: an arduous surgical access. Indian J Thorac Cardiovasc Surg.

[7] Esmaeil H., Al-Fadhli J., Dashti A., Al-Sarraf N. (2019). Ischemic mitral regurgitation in a patient with dextrocardia and situs inversus totalis. J Surg Case Rep.

[8] Kumano H., Shuntoh K., Yamaguchi A., Tatsu K. (2017). [Double valve replacement and tricuspid annuloplasty in a patient with situs inversus totalis and mirror-image dextrocardia; report of a case]. Kyobu Geka.

